# Beyond Messiaen’s birds: the post-verbal world of dementia

**DOI:** 10.1136/medhum-2018-011616

**Published:** 2019-05-29

**Authors:** Stuart Wood

**Affiliations:** Guildhall School of Music and Drama, London, UK

**Keywords:** dementia, music, care of the elderly

## Abstract

This paper investigates the use of verbatim musical transcription as a research method in dementia care. It reports on an art-based ethnographic study (Aesthetic Research in Everyday Life (Aeriel)) in which verbatim transcription was applied to everyday interactions in dementia care, making use of musical—instead of verbal—notation. Starting from the notion that medical and healthcare settings can be sites of ‘found performance’, the paper reviews literature relating to artistic methodologies within medical humanities, music, ethnography and dementia care. From this review, it proposes a research design and method of verbatim musical transcription as a potential avenue of investigating communication between carer and cared for in dementia care. The paper offers an illustrative example from Aeriel and draws conclusions from the synthesis of verbal and musical data analysis. Findings indicate an important advance in studies of dementia care communication towards a concept of the ‘post-verbal’ enabled by a musical research method and the clinical applications that it offers.

## Background

The Aesthetic Research In Everyday Life (Aeriel) study (Wellcome Trust UNS60836) was a qualitative art-based ethnographic study, using audio field recordings taken from dementia care settings, involving a verbatim transcription and thematic analysis which was based on musical, rather than verbal, listening. In other words, the study applied a musical ear to everyday dementia care communication in care homes by transcribing care interactions in musical notation based on their timing and tonal qualities as well as the words or vocalisations uttered. It asked how verbatim musical transcription of recorded dementia care communication can illuminate the often alienating world of dementia, concentrating particularly on what the study characterised as ‘post-verbal’ interactions. This novel concept relates to the individualised blends of verbal and vocal expression that are necessary to make meaningful and dignified connections with people living in later stages of dementia. Existing literature on nursing and care in dementia identifies deterioration in language as highly significant factors that often lead to anxiety both on the part of the carer and the cared for1. In nursing research, studies have claimed the efficacy of ‘singing’ in dementia care interactions without representing the musicality inherent in everyday communication with those living with deteriorating language ability.2–5 While child language development has long used musical framing to underscore theories of the preverbal,6 7 as yet, the expert musical ear has not been applied to inform an understanding of the post-verbal.

## Towards a musical methodology

What might it mean to listen to everyday dementia care communication as if it is music? Narrative explorations of musical events related to health, using social research methodologies, are common in medical humanities, with a concentration around evaluations of arts in health strategies8; yet, explorations of social events related to health, using a musical research methodology, are rare. Those that occupy related territories adopt a broadly ethnographic or sociological standpoint, such as the ethnographic accounts of music in mental health services from Ansdell and Denora,9 accounts of opera craft by Atkinson10 and a seminal work on listening in hospitals by Rice.11 Instead, art modalities are more often employed at the point of public engagement for the purposes of attracting attention or creating spectacle, or otherwise offered as a way of enhancing medical education. This paper will suggest that critical and enmeshed interdisciplinary enquiries into the value of a musical methodology for the medical humanities have a substantive, if novel, contribution to make. The research project Aeriel brings a musical research methodology to the topic of communication in dementia care.

This paper’s opening question extends the 20th century’s ‘found object’ and ‘found sound’ movements,12 prompting us to frame everyday life situations in care settings as already in a mode of performance and thus available for aesthetic analysis. In doing so, it also invites us to consider what research methods are available for such a task and how they might operate. The 20th century’s shift in awareness towards engaging with objects,13 sounds14 and later human actions15 as themselves containing equal agency to the viewer or listener resonates with parallel strands within current critical medical humanities. These strands explore the claims of agential realism,16 the turn to artistic research methodologies17and the prevailing interest in the post-human.18

While core works in child development and child psychology acknowledge the value of musical terminology in describing preverbal interactions,6 7 scant work has been done to advance what this paper will term ‘post-verbal’ interactions, such as those that occur with people living in later stages of dementia. Existing literature on nursing and care in dementia care consistently identifies this aspect of practice as highly sensitive and often leading to anxiety or alienation both on the part of the carer and the cared for.1 One significant factor in these reactions is the trauma inherent in the loss of verbal communication as a mode of creating meaning. Smaller pockets of research identify non-verbal communication19 to be a helpful means of alleviating that trauma. Music is a natural means of investigating this topic, because dementia care communication is rooted in non-verbal aspects such as rhythmic matching in vocal sound effects, pitch attunement in vocalisation, voiced signals of empathy and activity and the use of significant songs or musical fragments as orientating devices.20

The richness of interpersonal communication is difficult to investigate and report in words alone, even when concerned with standard language. Traditions of discourse analysis have demonstrated this through the development of alternative codes of notation, which are designed to convey not only the words spoken but also aspects of how those words were spoken and how the interaction was structured. This has already been applied to health communication, for example, in the case of GP consultations.21 Dementia care communication presents a unique everyday situation that arguably demands alternative or companion modes of representation (such as images, audio recordings or notation), but these alternative modes have yet to be critiqued in methodological terms. While performative and art-based strategies are becoming more accepted in social research, there is little discussion of what they can and can not offer to medical humanities as research methods, within an overarching aesthetic methodology and design. One exception, the systematic review by Fraser and al Sayah22 notes that the use of art-based methodologies in health research is very recent and requires considerable further refinement. The literature review below will explore musical research strategies across four related disciplines: medical humanities, music, ethnography and dementia care.

## Literature

### Medical humanities

No studies demonstrate a direct application of verbatim musical methods as a research methodology within medical humanities. Accounts exist of evaluation or reflection on musical practices in care settings or a medical context, but these exist in a very particular formation: social research methodologies applied to musical subject matter. Moss, Donnellan and O’Neill explore the notion of ‘aesthetic health’ in the context of older people’s hospitaliation,23 whereas Shakespeare and Whieldon evaluate a singing programme in the context of mental health.24 Moss and O’Neill develop their concept of aesthetics in medicine, proposing a notion of aesthetic deprivation.25 These examples maintain a social research focus on art experiences in the medical context. Another trend in the medical humanities literature is to explore the musical canon as an illustrative tool regarding medical issues, for example, in Durà-Vilà and Bentley’s account of Britten’s opera Peter Grimes as a means of exploring mental health.26 In the current paper, I suggest that we might rotate the standard formation of using social or literary research methodologies on musical events in care settings, to apply musical research methodologies on care events in social settings. This is a radical and transformative question for medical humanities, but one with a precedent in the world of musical composition itself.

### Music

According to the western canon of classical music, noted examples of transcription of everyday sounds or speech as a compositional approach begin at the turn of the 20th Century. Ravel’s Entre Cloches (1897) and La Vallee Des Cloches (1905) and Enesco's Carillon Nocturne Op18 No7 (1916) are examples of how musical material was generated out of micro-listening and analysis of the sounds of the everyday world—in these cases, the sound of bells. Messiaen’s famous period of bird song transcription followed some 35 years later, in the mid 1950s (2007). Decades later, Steve Reich adopted a verbatim strategy in relation to spoken word, for example, in The Cave, 1993, a work based on the Cave of the Patriarchs of Hebron. As part of the post-colonialist turn in South East Asia towards a focus on celebrating the local and indigenous cultures of the region, Singaporean composer Leong Yoon Pin used a verbatim method to capture the characteristic vocalisations of food sellers in his choral piece, Street Calls (1997). More recently as part of a verbatim theatre methodology, Blythe and Cork applied verbatim theatre methods to recorded interviews about a notorious Ipswich serial killer as material for their seminal musical, London Road (2011).

These works often explore aspects of communication (eg, in Messiaen’s birdsong works, Leong’s Street Calls and in London Road’s narratives) and overall demonstrate a broad concern with addressing sociopolitical or historical topics. It could be argued that these artistic works take a unique method of enquiry that is guided by an ethical stance towards the subject matter of the everyday. These works do not reduce their subject matter to mere informant or signifying object but instead elevate them to a generative role in which they are active collaborators in the process of knowledge production. In this way, the birds in the alps, food sellers of Singapore and residents of an Ipswich street are perceived in ‘found performance’: as a performative mode already in motion. Meaning is produced by meeting that performative mode with an artistic methodology. What meaning is produced, reinforced and materialised in that connection between the sound of everyday life and the epistemology of medical humanities or allied disciplines? It is perhaps that the world of the everyday is actively co-creative. When Reveil d’Oiseaux, Messiaen’s first major piece of birdsong, was first rehearsed, its note-perfect play through left him bitterly disappointed. The performance had no soul. No spirit. Only when his pianist Yvonne Loriod took a trip to the woods with her mother for inspiration was she able to breathe life into the piece. At the second rehearsal, the composer was in joyful raptures.27 Clearly, the woods helped.

The focus in this paper is on the methodological use of verbatim musical transcription, which is closely allied with verbal transcription, as used in ethnographic research. This literature review does not include dramatic or evocative musical moments that echo or appropriate everyday life. It would exclude, for example, the Music Nationalism movement of the 19th Century which transcribed folk song for compositional treatment. It would also exclude the incorporation of scene-setting traditional, religious or cultural musical forms in operatic or symphonic scores such as the use of traditional dance forms in Bizet’s Carmen (1875). Quotation from real life is also related but not part of this study, so that pieces such as Berberian’s vocal solo work Stripsody (1966) or Cage’s 4’ 33 (1942) are close neighbours but not part of this review.

### Ethnography

Verbal transcription is a standard technique in ethnographic research.10 Increasingly scholars are exploring methods of developing the basics of recording words or speech, via often art-based methods such as drawing or sound recording. In this search, no examples were found of studies where verbatim musical transcription was used as a methodology within ethnography. Practices of listening, recording and analysis using artistic methods do exist in sound-based ethnographic accounts of the medical setting. Rice, for example, provides a substantial account of listening in hospitals. In his text Learning to Listen, Rice28 notes how auscultation is based on the notion that ‘subtleties of pitch, rhythm and dynamics in a murmur express particular physiological changes’.

In their account of how sound is used in medical training, Harris and Van Drie describe three categories of capturing sound for the purposes of medical education: demonstration; mimicry and repetition; and rhythm and improvisation. They note the use of graphic scores in training recognition of arhythmic heart beats.29 Direct musical notation is not featured. In the 1960s, cardiologist Dr Harvey used symphonic recordings to introduce students to the principles and techniques of aural identification and sound classification. Students were taught, through repeated listening of the recordings, to isolate a particular instrument from the more general orchestral background. This would provide them with a context for turning to similar analysis and descriptions of heart sounds.30 Harris31 explores how medical practitioners hear and share sonic information by how they frame it as percussion. These can be viewed as a musical methodology of medical education. Ingold observes that ethnographers often make drawings in their notebooks but that these have rarely been considered methodologically or as ways of understanding more about the object of study. He argues that drawing allows a more direct and collaborative form of engagement in the field, which involves the researcher in what he calls ‘embodied notation’, making interpretations that attend to different rhythms of the environment than may be picked up by written words or sound recordings.32

Art-based research methods are valuable in situations where the topic of the research is difficult to put into words or to classify. Medicine has its own multiplicity, which is met by the methodologies of the humanities. This multiplicity might be described by Jane Bennet as ‘a vitality at work both inside and outside of selves’.33 Artistic methodologies help translate the multiplicity as it acts on us, often without needing to name it. That the unnamed can also be known involves what Ashon Crawley might call ‘a centrifugitive refusal of centeredness’.34 In his account of an aesthetics of vocality in black pentecostalism in the USA, Crawley muses on how in the vocalisations of black pentecostal congregations ‘the unspeakable is vibrated and sounded out…’34. The move from the unspeakable to the attempt to name is not a simple epistemological line. The history of auscultation and auditory diagnosis is a good example of why musical techniques remain relevant in that move.

In his recent comments on sound-based methods in ethnography, Atkinson35 argues that ‘[S]ociologists and anthropologists have not always been sufficiently alert to the aesthetics of their research settings’.36 He also notes how everyday activities contain performative elements that bear close scrutiny using appropriate research methods: ‘…ethnographers do need to pay attention to the forms of interaction, of social encounters and of social events…(these accounts) should also include proper attention to the enactment of performative encounters within the fabric of mundane social activity…’.37 Musical attention to everyday encounters in social settings is one established method of drawing out what Atkinson10 would call ‘their own temporal frames, their cycles of events, their daily round, their distinctive rhythms’.38 In his account of walking, Edensor39 draws on the ‘textworks’ of Richard Long, Francis Alys’s series of walks entitled Railings and Jeremy Deller’s 2009 Procession, along with the work of Lefebvre40 to illuminate his perspective that the rhythms and timing of everyday life bear ethnographic exploration. The application of this perspective in the medical field is not lost on Atkinson, who suggests that ‘[S]ound, its recognition and its assessment, forms a significant part in the organisation of medical knowledge and practice…[s]ound expands the clinical gaze…’.41 He also makes an argument that attunement to sound is available as a tool in the general ethnographer’s kit, arguing that while some researchers may identify themselves primarily as working in sound, or through the use of soundscapes, future ethnographers may need to regard such work as part of their standard kit of documenting cultural and social activity.

### Dementia care

Although music plays a significant part in aspects of dementia care research, it tends to appear as an arts in health practice or therapeutic intervention on which clinical or social research methods have been applied. No accounts exist of verbatim musical transcription being used as a central methodology in dementia care research. Speculations appear in some corners of music therapy literature on the musicality of everyday interaction.12 These exist as extensions of the theory of communicative musicality developed in relation to child development and ‘preverbal’ interaction. Stephen Malloch describes how he hit on the term ‘communicative musicality’ to describe the essence of parent–infant interaction:

As I listened, intrigued by the fluid give and take of the communication, and the lilting speech of the mother as she chatted with her baby, I began to tap my foot. I am, by training, a musician, so I was very used to automatically feeling the beat as I listened to musical sounds.… I replaced the tape, and again, I could sense a distinct rhythmicity and melodious give and take to the gentle prompting of Laura’s mother and the pitched vocal replies from Laura… A few weeks later, as I walked down the stairs to Colwyn’s main lab, the words ‘communicative musicality’ came into my mind as a way of describing what I had heard.42

This insight is echoed by Stern’s work on Vitality Affect in child development. Stern asks:

…why is it necessary to add a new term for certain forms of human experience? It is necessary because many qualities of feeling that occur do not fit into our existing lexicon or taxonomy of affects. These elusive qualities are better captured by dynamic, kinetic terms, such as “surging,” “fading away,” “fleeting,” “explosive,” crescendo,” “decrescendo,” “bursting,” “drawn out,” and so on.43

Dementia care relies similarly on expressive vocal components to build relationships and carry meaning, often taking precedence over the semantic content of words. For example, dementia care communication is influenced significantly by the impact of emotional tone. Willams and Herman44 demonstrate that emotional tone is an influencing factor in creating resistance to care in dementia care settings. Ward *et al* 5 also make a strong case that communication is a contested area of dementia care. The authors claim that communication is at the heart of person-centred dementia care, and yet people with dementia can be excluded from person-centred care through inappropriate communication methods. They also suggest that medical education and care training should reflect better approaches to dementia care communication. This claim is also at the core of Kitwood’s interpersonal approach to dementia care.45 Research that explores the role of music in communication allows a technical and non-judgemental pathway into discovering modes of varying emotional tone, and reaching close interpersonal attunement, through a focus on musical parameters such as pitch, speed or dynamic.

This notion is introduced in a series of papers on singing in nursing and caregiving as part of dementia care. A significant allied field in relation to the musicality of dementia care communication is the literature on ‘music-therapeutic caregiving’. Brown *et al* 2 argue for the necessity of music making as part of clinical care. This signposts a body of literature on the impact of caregivers’ singing in relation to a range of everyday events in dementia care, including the use of background music in social situations,46 use of voice to help with person transfer moments,47 singing during morning care routines48 and the role of music in transcending the limits of institutionalisation.49 Apart from the presence of music making in care interactions, research also points to the value of musical environments in dementia care50; the impact of music therapy on language function in dementia care51; the effect of music in the alleviation of apathy among people living with dementia52; the case for improved clinical outcomes using non-pharmacological methods53; the management of behavioural symptoms of dementia using music54 55 and the impact of music making on staff attitudes towards people living with dementia.56

Although music arguably has a clinical and recreational value in dementia care for its role in building meaning and promoting relationships, it has yet to be used as a core method of research for analysing everyday speech or care interactions that are not characterised as explicit, directed musical events. In other words, research is yet to approach everyday care interactions as ‘found performance’ and use a methodology rooted in the humanities to illuminate it. One reason for this may be that verbatim musical transcription in this context requires a specific skill set in the researcher. Musical notation can be perceived as an arcane and elitist strategy especially when applied in the setting of care homes or dementia care hospital wards. Dementia care settings are equally hard to reach for many researchers, as they are protected safe places for vulnerable people and yet are also conditioned to a culture of inspection and anxiety. The art-based research that does find a place in dementia care tends to be via evaluation of professional musical interventions, within the music therapy or Arts and Health frame. This may be in part due to the professional status of music therapy research and practice and the perceived validity of music practice in dementia care.

## Research study method and design

The guide question for the Aeriel study can be summarised in this speculation: what is the musicality of everyday dementia care communication? The study did not aim to provide an apologetics for the musicality of people living with dementia, but rather to trial a musical methodology for documenting a whole care situation. This aim counts the care home resident, carer and the wider soundscape as elements for inclusion within the research methods. The project design involved audio recording of everyday care home interactions (personal care, mealtimes or social interactions) in three care homes, using small digital audio recorders; a selection process to identify indicative examples and then verbal transcription of those selections in full, followed by an open verbal coding and classification into descriptive themes. This process also created a shortlist from which selected musical transcriptions were derived as finished scores. No public performance of musical material was included in the project design. Recruitment involved first contact via the Director of Nursing of a national care home company, then personal meetings with the General Managers of three care homes and their regional managers. The direct approach to potential participants took place via a liaison staff member in each home, after preparatory meetings to clarify research schedules and data security. The full data set comprised 18 audio tracks, totalling 2 hours 20 mins of recordings.

In care homes, the particular factors of intricate staff rotas, unpredictable changes in staff and resident availability, the fears and anxieties associated with research intrusion and the often continuous traffic of staff training, inspection and visitation all add complexity to a research design. Care homes often have their own unique characteristics and moods, their own daily pace and their own power channels or organisational psychology around observation and criticism. They have unique concerns around how to manage commercial issues such as marketing or PR in relation to vulnerable people. This constellation of factors suggests that an internal liaison figure would be valuable in order to ensure data security, provide knowledge of staff rotas and lend credibility to the requests inherent in a project of this type. Of similar importance is a senior gatekeeper, who would provide access, institutional credibility and risk management accountability.

There are rightly stringent ethical and practical requirements in this setting, not only around data security and safeguarding but also around the public use of musical material generated by the transcription and composition process. Given the current culture of social media and marketing related to dementia care, it is natural that potential participants would be concerned not only about the safety of their residents and care communities but also about the risk of the researcher making commercial gain out of music featuring their recordings, or written about them. The Aeriel ethics process involved a three-stage approval protocol designed to provide clear information on these concerns. This included research ethics committees at the affiliated research host and the hosting private care home company, concluding with an on-site ethics check with care home management. Participants were informed that the purpose of the recording was to transcribe their interactions musically, and that those transcriptions and selected recordings might be shared in an academic context. It was expressly set out that this was not theatre Research and Development, material for public musical performance, or something I would write songs or musical works about. In some cases, we gained full informed consent to record from Next of Kin, in a protocol that was led and kept under review by a trusted staff liaison rather than by the lead researcher. The data gathering and analysis protocols were designed to reflect the complexity of both the practical setting and ethical considerations described above.

## Findings

The study involved two strands of data analysis: a verbal coding of musical aspects of communication and a musical analysis in the form of verbatim scores. The findings are presented here through a selected example of a mealtime interaction in a care home, between a care worker and a resident.

### Research study example

**Figure 1:**
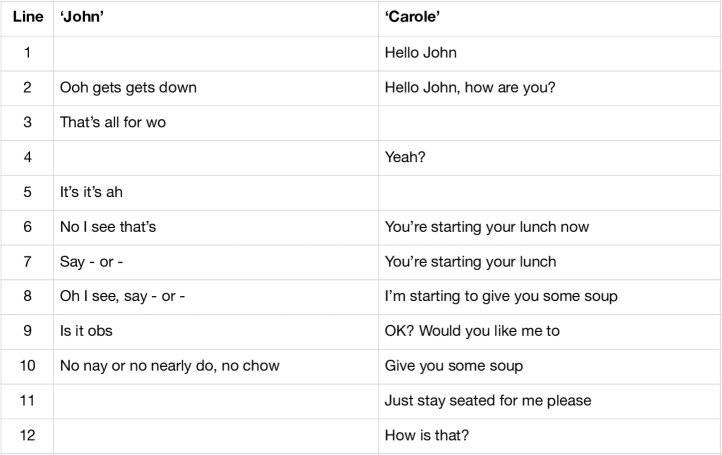
Extract of verbal transcription of mealtime encounter.

In the dining room of a care home, healthcare assistant ‘Carole’, assists ‘John’ with his lunch. In the selected verbal transcription below, we see Carole greeting John, who then speaks with a series of seemingly broken and interrupted syllables. Carole proceeds to frame the moment by saying that lunch is starting and that she has soup for John to try. She speaks over his broken speech, perhaps to help settle or orientate him in this moment of transition (Figure 1).

**Figure 2:**
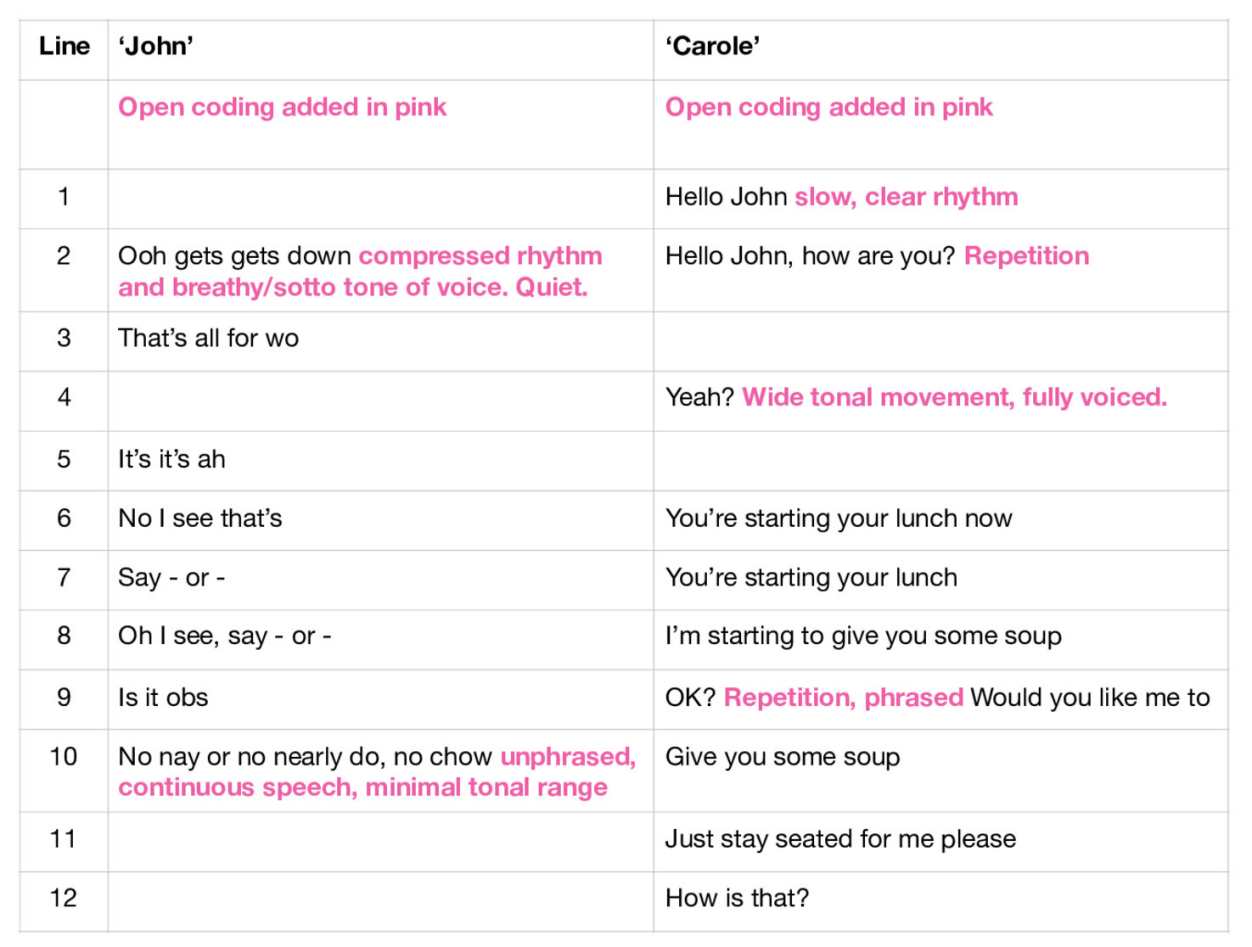
Open coding of extract of verbal transcription of mealtime encounter.

To reflect the communicative scenario that takes place within, through and beyond the words spoken, I made a verbal open coding using musical terminology, shown here in pink. I describe, for example. the airy quiet of John’s vocal tone (sotto tone of voice) and how the restless feeling of compression in the timing of his speaking (compressed rhythm) contrasts with the (slow clear) rhythm of Carole’s diction. I note in this coding how the pitch (tonal) range of John and Carole seems different: minimal in John’s speech, wide in Carole’s. This was a cyclical analysis process, and involved numerous returns to the recording and the codes. At this point, using audio compositional software, I also adopted a listening strategy of seeking a pulse within John and Carole’s speech separately that would provide a starting point for writing it down. I found that I heard John’s vocalisations most clearly when I set a metronome pulse at 120 bpm, which means that his rhythms of speech were most easily reproduced when I applied that pulse as a listening framework. I found Carole’s speech was most easily notated when I heard it at 92 bpm (Figure 2).

On classifying the codes derived from all the extracts, I removed the references to whether they came from the analysis of residents’ or carers’ speech. Aeriel is not designed primarily to produce generalised knowledge demonstrating that the speech of people living with dementia is musical, but instead to explore what musical categories of experience are co-created between people interacting in dementia care settings.

Six musical categories of experience emerged from the coding and classification:

- Interactional structures.
- Soundscape.
- Topic/theme.
- Speed/rhythm.
- Phrasing.
- Tone of voice.

**Figure 3:**
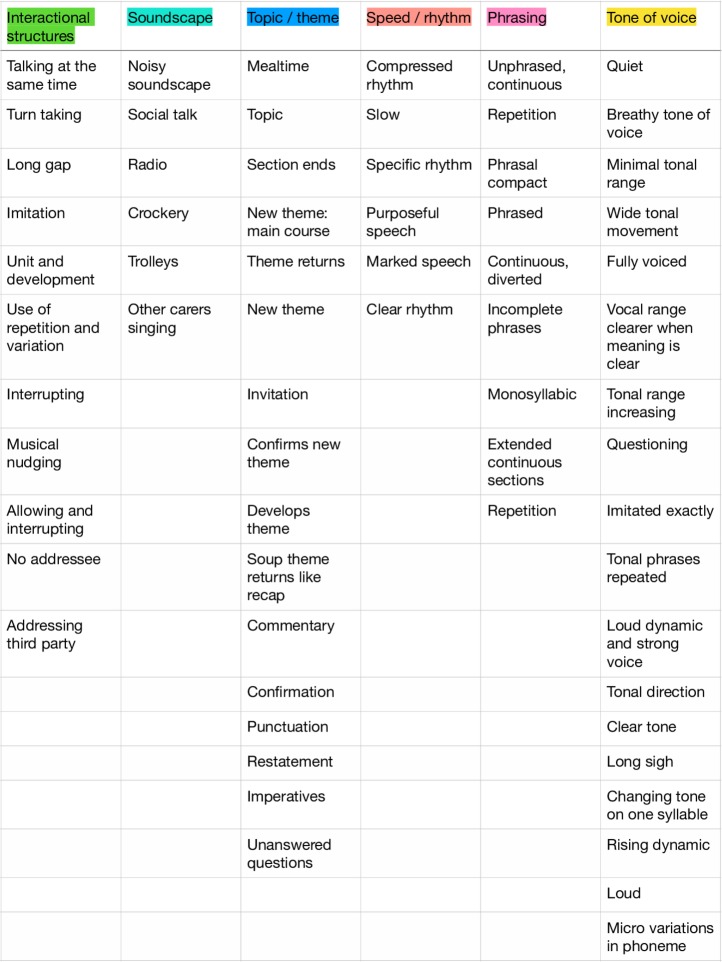
Six categories of musical experience emerging during mealtime.

The codes are shown in Figure 3 under the headings of the six categories.

These categories illuminated the basic musicality which arguably constructs meaning between John and Carole. For instance, the analysis characterises the interactional structures by which they are able to maintain a conversational to and fro, despite interruptions, simultaneous utterances or long gaps. The analysis also draws out the soundscape of everyday mealtimes against (and possibly, despite) which John and Carole interact. As some of their verbal content is topical and related to the concrete moment of a mealtime, there are pervasive aspects of topic, or theme, and their development. In terms of the materiality of their vocalisation, the speed, rhythm, phrasing and tone of their voices is rich in musical information. For example, the analysis registered characteristic phrasal patterns that included continuous seemingly unphrased speech, compact forms, broken or incomplete phrases, monosyllabic units and phrasal repetition. These elements of interaction and phrasing produced rich codes and examples even in moments that involved non-standard grammar, instances of word loss or compressed broken speech.

The next stage involved musical transcription of selected moments. Selections were made on the basis of how clearly they illustrated aspects of the verbal coding such as characteristic phrasing, or how they demonstrated methodological complexities of pitch recognition, transcribing natural speech rhythms or layering speech over background radios and the percussive sounds of crockery or trolleys. At its structural level, musical transcription involves two primary choices: time values and pitch values. These are the most significant aspects also that verbal transcription is unable to communicate.

**Figure 4:**
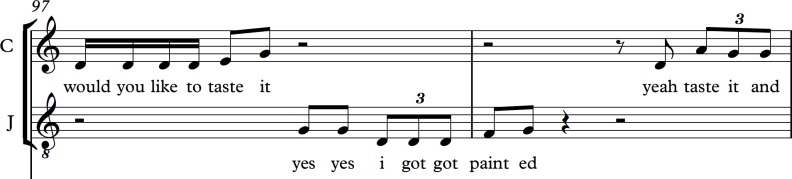
Musical score excerpt.

In Figure 4, the timing and tonal direction of their communication is laid out in musical notation. Note how the rising tone in Carole’s ‘would you like to taste it?’ precedes a similar inflection from John. John’s words suggest he mishears what Carole said, or that the effects of his dementia have caused an aphasic word substitution. His timing and vocal pitch, however, suggests a personal alignment with Carole that is not apparent from reading the spoken words alone.

## Discussion

### Post-verbal communication

In musical transcription, a person’s characteristic phrase lengths, rhythmic habits and unconscious timings are revealed as important signatures of personhood. John’s vocal tendencies include a natural rolling ‘triplet’ figure, which contains a variety of linguistic formations such as, ‘I got got’ in the example above. While the semantic integrity of John’s speech is shown in these extracts to be diminished, this study suggests that musically expressive components remain embedded in a vocal ability that has developed and been practised over seven decades. John’s prosody and vocal rhythm convey a personhood that emerges despite the limiting effects of dementia. He is, arguably, entering a state that is not ‘non-verbal’, but ‘post-verbal’. In this state, his musical expression may not have communicative equivalence to his pre-existing vocal acuity but it gives access to those around him to engage as equals vocally with him. As healthcare professionals, it may be beneficial to pay close attention to the rhythm and indeed the pitch of a person’s vocalisations in order to honour and enhance the personhood that will have been attributed to them across their life until now. Musical listening in later stages of dementia affords a personhood that risks being lost when verbal abilities decline. For her part, Carole’s vocal tendencies display a mannered awareness of phrasing seemingly designed to engage at a post-verbal level while performing at an acceptable level of social decorum. She speaks in friendly but short clips, intended perhaps to convey warmth but also to elicit participatory vocalisation from John. This is heard in her repetition of phrases: ‘would you like a taste?’ and her platitudes: ‘OK alright then’. When developed as musical notation, the rhythms of John’s prosodic tendencies and Carole’s professional register are enhanced and brought respectfully into the light.

One assumption of this study was that a person’s vocalisations contain not only rhythm but also pitch. This does not mean that every utterance is heard as if sung out in full, but simply acknowledges that vocalisations require a frequency that is produced by the vibration of vocal folds in the larynx. In some moments, the vibration produces clear tones, even when they are unintentional. This can often happen during vocal effects such as laughter or surprise, but also unwittingly during normal conversations. Note, when you next pause in speech, the tone in which you say, ‘erm…’ (everyday you sing when you don’t know what to say). At other times, vocal pitch is suggested, approximated or involves sliding directions of tone rather than static held pitch. What emerges in musical transcription are the moments when John and Carole’s speech is precisely imitative and convergent in tone and rhythm, even when the verbal transcription may convey divergence in understanding and semantic meaning.

In verbal coding, it is possible to write down every phonic sound in text, whether that is in standard English or not, and it is possible to write down the chronological order of its interaction. Existing traditions of discourse analysis also allow for the representation of expressive aspects such as interruption and simultaneous speech. In musical coding, the interaction adopts an integrated semiotic system that offers a form of shorthand echoing the preverbal theories of Trevarthen and Malloch6 and Stern.7 This integrated semiotic system presents the blend of verbal and musical meaning within a simultaneous visual form. This form delivers representational signs (‘time for your lunch’) with a vocal presencing bound up in showing the timing and tone of voice and still more subtle manifestations of warmth or fatigue through tonal direction or repetition for example. The findings suggest that a musical research methodology occupies a territory in which registers of knowledge interact. A musical methodology is a mode of entanglement which makes use of seemingly objective matters (such as speed of speech or vocal frequency) and emergent forms of aesthetic embodiment.

### Multiple time frames

**Figure 5:**
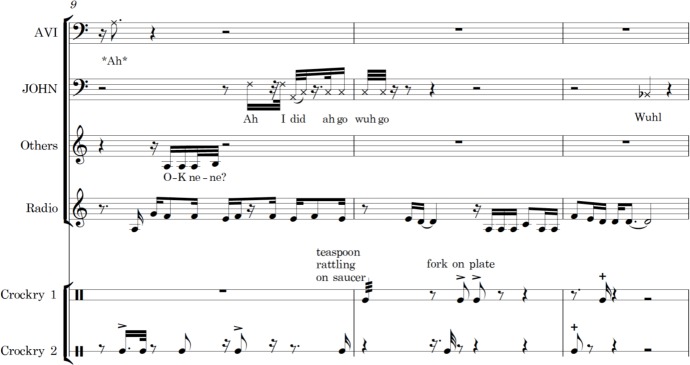
John and Avi with background sounds of radio, crockery and other staff.

A further property of musical structure is that it can convey multiple time frames at the same time. Musical transcription presents the timing of micro-repetitions of speech within the broader cycles of returning topics, periods of eating or institutional flows. This was laid out musically in the analysis phase by constructing larger scores that included not only vocal lines but also percussion lines for cutlery, trolleys and radio. In Figure 5, a different score shows ‘John’ being assisted on another occasion by ‘Avi’. Here, we also see notation of the sound of a third person in the background, the radio playing and layers of crockery (abbreviated to Crockry in the example) as percussion.

These transcriptions produced scores that resembled chamber music, for 6–10 parts in some cases. The scores show how micro-moments of repetition are played out against the backdrop of longer cycles: songs on the radio playlists; a carer’s cycles of assisting one or more residents and of the care setting’s own institutional patterns of mealtimes and cleaning. Lefevbre’s40 attention to the rhythmic cycles of everyday social life are manifest here in a directly visual mode of representation.

## Clinical applications

This research methodology offers creative applications for thinking about care communication more broadly. It espouses a stance of non-judgmental listening and of attending to the structures of communication rather than focusing only on verbal content. Aeriel could, for example, provide a non-judgmental entry into exploring moments when communication between caregiver and care recipient are more difficult, by encouraging a practice of listening not only to the verbal content of speech but to the tone, timing and phrasing of those encounters. It could generate a mutually person-centred care approach by valuing the musical sensitivity inherent in carers’ communication and giving them permission and opportunity to nurture that quality. It could add to the increasing practice of raising sensitivity to soundscape, background noise and music for mood setting within care environments. It could also complement mainstream research methodologies and inform the evidence base for music therapy for those with cognitive impairment. The implicit focus on performativity at the heart of this study also offers avenues of role play which can enhance medical and care-based trainings. As suggested earlier in this paper, an often unacknowledged factor in everyday care situations is the well-being of the carer. The trauma of the loss of verbal language in the person with dementia is often passed on to the carer. Aeriel potentially lends a more refined listening ear to both sides of the care dyad as equals.

## Conclusion

There are as yet no examples in the literature of verbatim musical transcription being used as a method of research in medical humanities within an aesthetic methodology. It is arguable, however, that an interdisciplinary approach to this research method would address the most commonly cited issues within the field, contributing to discussions of definition, developing methodological approaches and relocating the clinical position of the arts. It would offer an original entry point for medical education and an important perspective on medical ethics. In doing so, it creates questions around research ethics, particularly patient access, patient benefit and consent that can most effectively be addressed from an interdisciplinary perspective. Possibly, this method is best supported by a research approach that is rooted in the art therapies and that embraces ethics of care informed by the new frontiers of ethnography.

The sound environment and the practice of listening as a research method are novel but accepted topics within ethnography, but the field has only recently been encouraged from within to pay closer attention to the modes of notation that are used in that research subdiscipline. The established field of sound research within healthcare and medicine would be enhanced by further refinement of musical listening as a research method. This is of course a technical craft and currently one that is dependent on a knowledge of musical notations, softwares and musical traditions, which can be seen as a limitation of the methodology under review here. However, this pilot of verbatim musical transcription offers a significant step towards making musical notation more available to a general ethnographic practice. It could also be argued that this skill might be a useful addition to the medical toolkit: should all doctors learn to write music?

This method goes way beyond Messiaen’s birds. There are natural ethical implications for the appropriation of musical composition as a research strategy. Musical works differ from traditional text-based research narratives in that they generate affective responses via their inherent structures. This can risk drawing the work into over-representing or indulging the voice of the author or performer. It could be argued that standards of research validity that would be normally applied in qualitative research are less easily applied to musical compositions presented as interdisciplinary research, even when they are positioned within a sociopolitical or historical frame. I would argue that research ethics apply and map directly at the stages of project design, setting up, field access, data gathering and analysis and in data management and protection.

It may be that in occupying the interdisciplinary platform of musical social research, new aspects of research ethics will emerge in the process. Indeed, themes drawn from this mode of research relating to the risk of manipulation or ambiguity of meaning, intellectual property rights over artistic works, issues of dissemination and appropriation of voice could well feed instructively back into the mainstream context of medical humanities research. Performance or artistic research outputs sit ambiguously in the field of medical humanities, often being modes both of data processing and also of public engagement. The emerging voices of ethnic studies,57 socially engaged art performance and drama58 59 may further inform ethical and practical rigour in a turn towards ‘found performance’ in the medical humanities. Verbatim musical methods may be particularly helpful in that turn.

## Notes

1. Eva Eggenberger et al. (2013), “Communication Skills Training in Dementia Care”

2. Steven Brown et al. (2001), “‘Music-therapeutic Caregiving’: The Necessity of Active Music-making in Clinical Care.”

3. Eva Götell et al. (2002), “Caregiver Singing and Background Music in Dementia Care.”

4. Lena M Hammar et al. (2011), “The Impact of Caregivers’ Singing on Expressions of Emotion and Resistance During Morning Care Situations in Persons with Dementia: An Intervention in Dementia Care.”

5. Richard Ward et al. (2008), “A Different Story: Exploring Patterns of Communication in Residential Dementia Care.”

6. Steven Malloch and C Trevarthen (2009) , *“Communicative Musicality. Exploring the Basis of Human Companionship*.”

7. Daniel Stern (1985), “*The Interpersonal World of the Infant*.”

8. Hilary Moss et al. (2012), “A Review of Qualitative Methodologies Used to Explore Patient Perceptions of Arts and Healthcare.”

9. Gary Ansdell and Tia DeNora (2017), “Music in Action: Tinkering, Testing and Tracing Over Time.”

10. Paul Atkinson (2017), “*Thinking Ethnographically*.”

11. Tom Rice (2013), “*Hearing the Hospital: Sound, Listening, Knowledge and Experience*.”

12. Stuart Wood (2017), “Found Performance: Towards a Musical Methodology for Exploring the Aesthetics of Care.”

13. Margaret Iversen (2004), “Readymade, Found Object, Photograph.”

14. Richard Kostelanetz and John Cage (1987), “The Aesthetics of John Cage: A Composite Interview.”

15. Klaus Groh (1981), “Performance Art: What Is It?.”

16. Karen Barad (2007), “*Meeting the Universe Halfway: Quantum Physics and the Entanglement of Matter and Meaning*.”

17. A Mermikides et al. (2016), “*Performance and the Medical Body*.”

18. Luna Dolezal (2017), “Representing Posthuman Embodiment: Considering Disability and the Case of Aimee Mullins.”

19. Wilma M Caris-Verhallen et al. (1999), “Non-verbal Behaviour in Nurse–Elderly Patient Communication.”

20. Dorothy Miell et al. (2005) , *“Musical Communication*.”

21. Christian Heath (1986), “*Body Movement and Speech in Medical Interaction (Studies in Emotion and Social Interaction*).”

22. Kimberly Diane Fraser and Fatima al Sayah (2011), “Arts-based Methods in Health Research: A Systematic Review of the Literature.”

23. Hilary Moss et al. (2015), “Hospitalisation and the Aesthetic Health of Older Adults,” 10.

24. Tom Shakespeare and Alice Whieldon (2018), “Sing Your Heart Out: Community Singing as Part of Mental Health Recovery”.

25. Hilary Moss and Desmond O'Neill (2014), “Aesthetic Deprivation in Clinical Settings.”

26. G Durà-Vilà et al. (2009), “Opera and Madness: Britten’s Peter Grimes—A Case Study.”

27. C Dingle (2007), “*The Life of Messiaen*.”

28. Tom Rice (2010), “Learning to Listen: Auscultation and the Transmission of Auditory Knowledge,” 46.

29. Anna Harris and Melissa Van Drie (2015), “Sharing Sound: Teaching, Learning and Researching Sonic Skills.”.

30. Melissa Van Drie (2013), “Training the Auscultative Ear.”.

31. Anna Harris (2016), “Listening-touch, Affect and the Crafting of Medical Bodies Through Percussion.”

32. Tim Ingold (2013), “*Making: Anthropology, Archaeology, Art and Architecture*.”

33. Jane Bennett (2009), “*Vibrant Matter: A Political Ecology of Things*,” 62.

34. Ashon Crawley (2017), “*Blackpentecostal Breath: The Aesthetics of Possibility*,” 145.

35. Paul Atkinson (2015), “*For Ethnography*.”

36. Paul Atkinson (2015), “*For Ethnography*,” 109.

37. “Paul Atkinson (2015), “*For Ethnography*,” 76.

38. Paul Atkinson (2017) , *“Thinking Ethnographically*,” 154.

39. Tim Edensor (2010), “Walking in Rhythms: Place, Regulation, Style and the Flow of Experience.”.

40. Henri Lefebvre (2004), “*Rhythm Analysis*.”

41. Paul Atkinson (2015), “*Thinking Ethnographically*,” 140–141.

42. Steven Malloch and C Trevarthen (2009) , *“Communicative Musicality. Exploring the Basis of Human Companionship*,” 3–4.

43. Daniel Stern (1985), “*The Interpersonal World of the Infant*,” 54.

44. Kristine N Williams and Ruth E Herman (2011), “Linking Resident Behaviour to Dementia Care Communication: Effects of Emotional Tone.”

45. Tom Kitwood (1993), “Towards a Theory of Dementia Care: The Interpersonal Process.”

46. Eva Götell et al. (2002), “Caregiver Singing and Background Music in Dementia Care.”

47. Eva Gotell et al. (2012), “Can Caregiver Singing Improve Person Transfer Situations in Dementia Care?”

48. Lena M Hammar et al., 2011, “Impact of Caregivers’ Singing on Expressions of Emotion and Resistance During Morning Care Situations in Persons with Dementia: An Intervention in Dementia Care.”

49. Theresa Allison (2012), “Transcending the Limitations of Institutionalisation Through Music.”

50. Hans Ragneskog et al. (1996), “Influence of Dinner Music on Food Intake and Symptoms Common in Dementia.”

51. M Brotons and Susan Koger (2000), “The Impact of Music Therapy on Language Functioning in Dementia.”

52. Clive Holmes et al. (2006), “Keep Music Live: Music and the Alleviation of Apathy in Dementia Subjects.”

53. Kali S Thomas et al. (2017), “Individualised Music Programme is Associated with Improved Outcomes for U.S. Nursing Home Residents with Dementia.”

54. Farooq Khan and Martin Curtice (2011), “Non-pharmocological Management of Behavioural Symptoms of Dementia.”

55. Kendra D Ray and Mary S Mittelman (2017), “Music Therapy: A Nonpharmacological Approach to the Care of Agitation and Depressive Symptoms for Nursing Home Residents with Dementia.”

56. Huei-Chuan Sung et al. (2011), “Exploring Nursing Staff’s Attitudes and Use of Music for Older People with Dementia in Long-term Care Facilities.”

57. Ashon Crawley (2017) , *“Blackpentecostal Breath: The Aesthetics of Possibility*.”

58. Amanda Stuart-Fisher (2011), “Trauma, Authenticity and the Limits of Verbatim.”

59. James Thompson (2015), “Towards an Aesthetics of Care.”
